# Supplementary material for: Tumour-on-chip microfluidic platform for assessment of drug pharmacokinetics and treatment response
Source: Commun Biol. 2021 Aug 24;4:1001. doi: 10.1038/s42003-021-02526-y (PMC8385015; doi:10.1038/s42003-021-02526-y)
Supplement: Supplementary file 2 — Supplementary Information [file 42003_2021_2526_MOESM2_ESM.pdf]

Tumour-on-chip microfluidic platform for assessment of drug pharmacokinetics and treatment response

Supplementary Figure 1. SN38 and AZD0156 efficacy in 2D and 3D static setup
Treatment efficacy at 7 days using SN38 and AZD0156 as mono or combotherapy (with a fixed AZD0156 concentration) is illustrated in the following graphs. All experiments were carried on in a plate format, using regular 96 well plates for 2D setup and low-adherence 96-well plates for 3D setup. For both compounds, a 9-point dose-response was considered. SN38 concentration ranged between 0.01-100nM while for AZD0156 the range was 0.1-1000nM. Compound dispensing was performed by an HP3000 automatic dispenser. For combination schedules, AZD1056 fixed concentration were of 10 and 30nM respectively. Viability was evaluated by CellTiterGlo assay, as described in Methods.

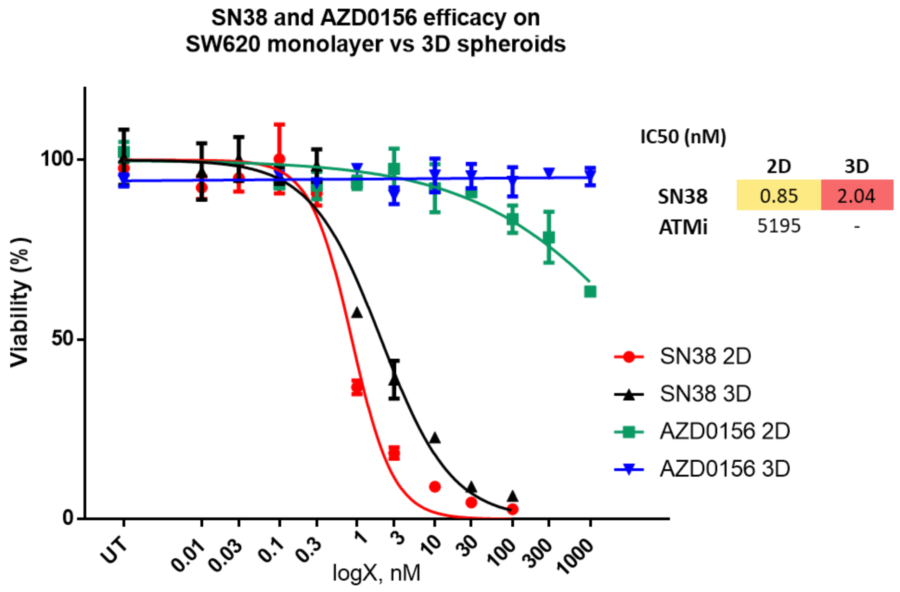

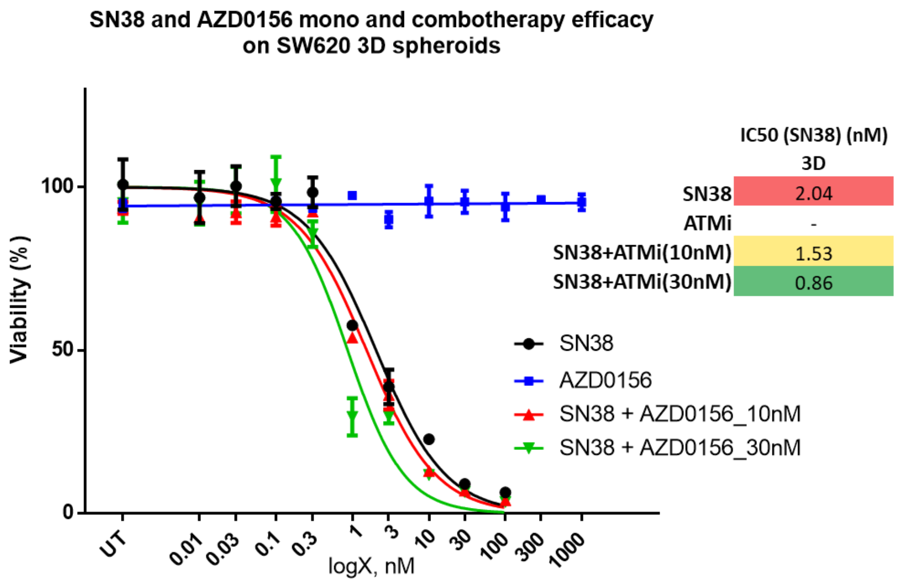

Spheroid size and morphology shows no effect for AZD0156 on SW620 spheroids at 7 days from treatment onset.

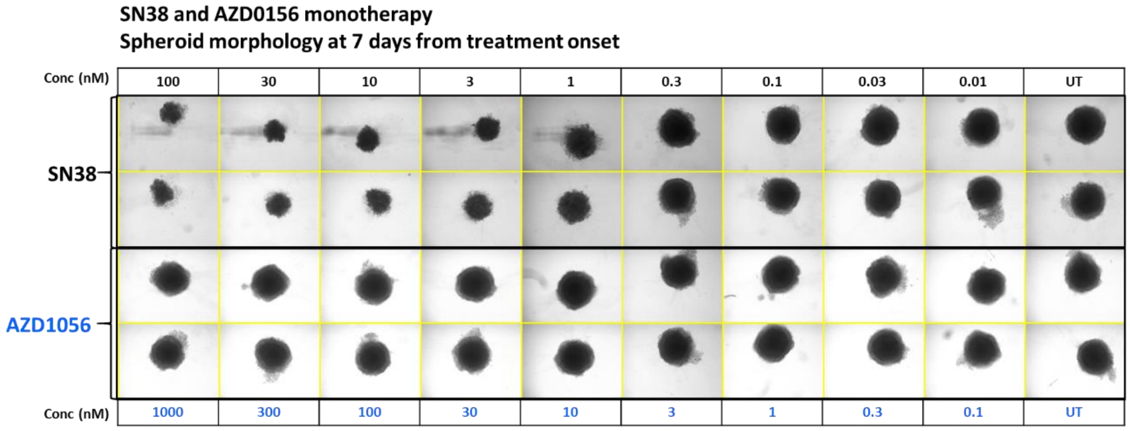

Supplementary Figure 2. Fluorescence image processing, per biomarker, using Fiji ImageJ custom-made macros
Biomarker quantitation

Each of the investigated biomarkers displays different morphologic particularities and location within the cells in the spheroid, so we had to design tailored macros to be able to perform a semi-quantitative analysis. Biomarkers detected within current experiments were γH2Ax, CC3 and Ki67. For all 3 biomarkers (γH2Ax and CC3), we have performed normalization by total Hoechst area.

γH2Ax location is nuclear, this biomarker being displayed as foci. To accurately quantitate this biomarker, it is important to be able to count foci (or cells containing foci) and to normalize this count with the cell volume (projection surface). Normalization is important as the spheroid volume and cell proliferation within the spheroid depends on the treatment type and schedule.

Quantitating these foci while imaging spheroids with a 20x objective can be tricky, especially when multiple spheroids are being imaged in a round-bottom well plate, in a volume of 50µL PBS, due to shadows and background noise effect. Thus, the macro we designed for this biomarker was able to count the number of γH2Ax positive cells in a spheroid, rather than counting all foci on all focal planes contained in an image batch.

Counting foci in individual images in a batch is prone to counting errors, due to factors as blurring effect during imaging foci with the 20x objective, in consecutive images in the same batch. Thus, counting individual foci in individual images in a batch can count the same foci multiple times, and in an inconsistent manner; the same time the foci measurement in order to define it as a particle to be counted is tricky and prone to errors.

<table>
<tr><td>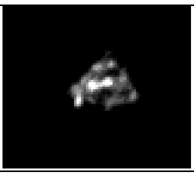</td><td>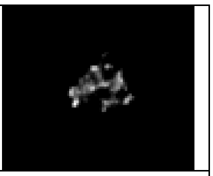</td></tr>
<tr><td>Image well A2
Slice 6</td><td>Image well A2
Slice 7</td></tr>
<tr><td colspan="2">γH2Ax foci</td></tr>
</table>

For a better γH2Ax quantitation, we have designed a macro that is analysing only the positive cells (nuclei) for this specific biomarker. As foci in a positive cell are clustering in the nucleus, they can be detected and then digitally dilated to get confluent, generating a γH2Ax "positive" cell. These "positive" cells are easier to be converted into countable particles. The macro we have designed is analysing the projection of all the slices/images in a batch for each individual spheroid rather than counting individual foci in each image in the batch, thus reducing counting errors among different experimental repeats. Foci counting variability among experimental repeats can be due to different spheroid placement in the imaging round bottom well plate.

To detect and count γH2Ax positive cells, following steps included in the specific macro.

1. all images in a batch for the "green" channel, corresponding to γH2Ax detection were "flattened" in a maximum intensity projection procedure;

2. the 8-bit projection image was binarized, using a predefined threshold (Intermode dark);
3. resulted spots were dilated 4 times, so the foci will get confluent to form the “positive cell”;
4. Count particles defined as positive cells (particles smaller than 300 sq pix were ignored, being considered as “noise”);
5. Particle (positive cells) count was normalized by total Hoechst area for the same spheroid. Total Hoechst area is described separately.

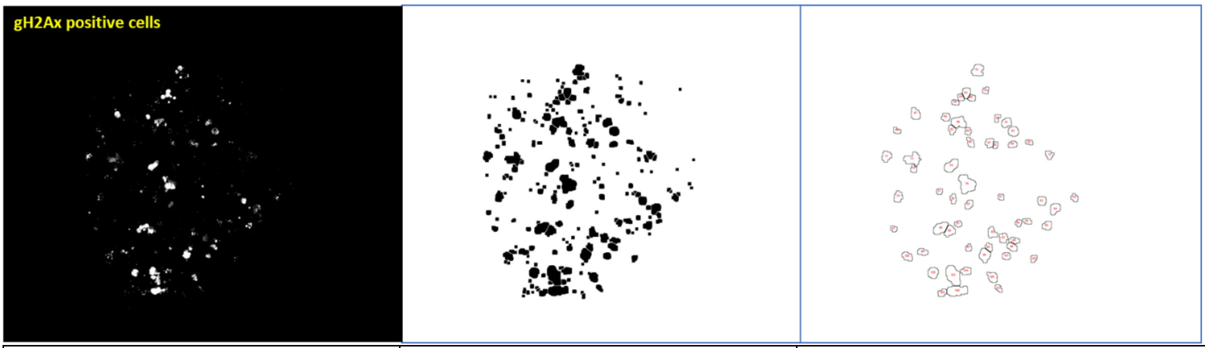

Max Int Projection from all images per channel per spheroid per well<br>No image correction<br>setAutoThreshold(“Intermodes dark")<br>run("Convert to Mask");	run("Dilate") 4 times so the small dots connect to form the “positive cell”	Count positive cells (particles smaller than 300 square pixels ignored from count

Images in the next montage illustrates and example of γH2Ax “positive” cell detection and count, using the custom-made Fiji ImageJ macro. One spheroid, treated by SN38 monotherapy SN38 (for 24h, PK profile) was fixed on day 7 from treatment onset, labelled for all biomarkers, transferred in the well A2 of an ultra-low adherence plate (Corning 7007), and imaged on CV7000 confocal microscope. Image batch for “green” channel, corresponding to γH2Ax positive cells was analysed using the specific GreenChannelMacro. Three different slices in the image batch are being represented in the montage (1, 18, 22). Positive cell count delivered by the macro was of 9 cells. This value was normalized further by total Hoechst area for the spheroid in well A2, using data from the “blue” channel.

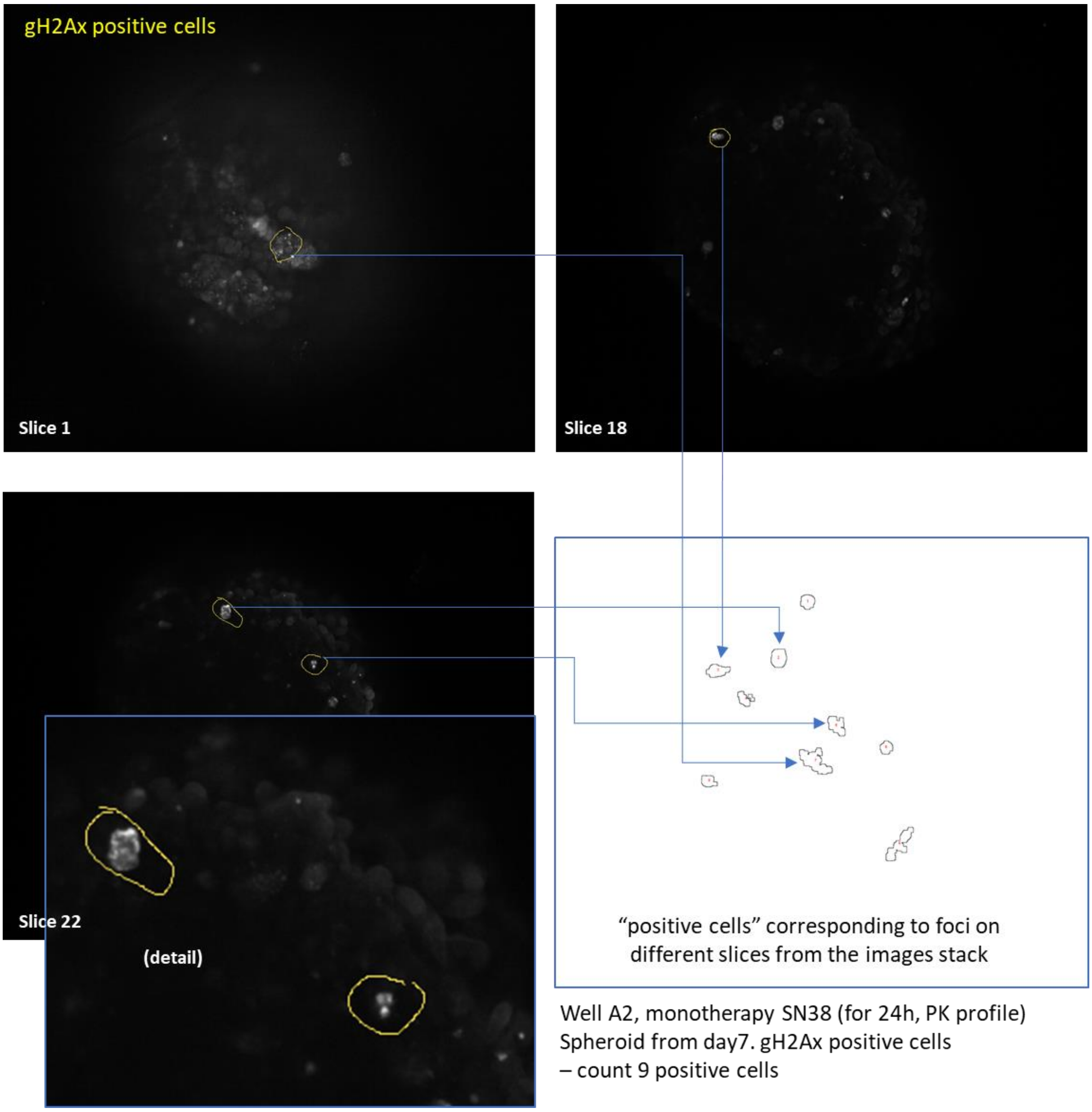

Well A2, monotherapy SN38 (for 24h, PK profile)
Spheroid from day7. gH2Ax positive cells
– count 9 positive cells

Total Hoechst area represents a parameter used for normalization within biomarker quantitation. This parameter represents the total measured area of the maximum intensity projection for a specific spheroid on the "blue" fluorescence channel, accounting for Hoechst staining (nuclear stain). Stained nuclei are detected as bright spots; the "Dilate" and "Fill Holes" functions are generating a large particle, associated with the total spheroid projection area. The procedure considers the following steps, gathered in a separate macro (Hoechst area macro).

1. all images in a batch for the "blue" channel, corresponding to nuclei detection were "flattened" in a maximum intensity projection procedure;
2. the 8-bit projection image was binarized, using a predefined threshold (Huang dark);
3. functions "Dilate" and "Fill Holes" were applied sequentially, so the spots representing the nuclei to get confluent in a large particle;
4. Measure particle area (results in squared pixels);
5. Total Hoechst area measurement result is used to normalize the three detected biomarkers for the same spheroid.

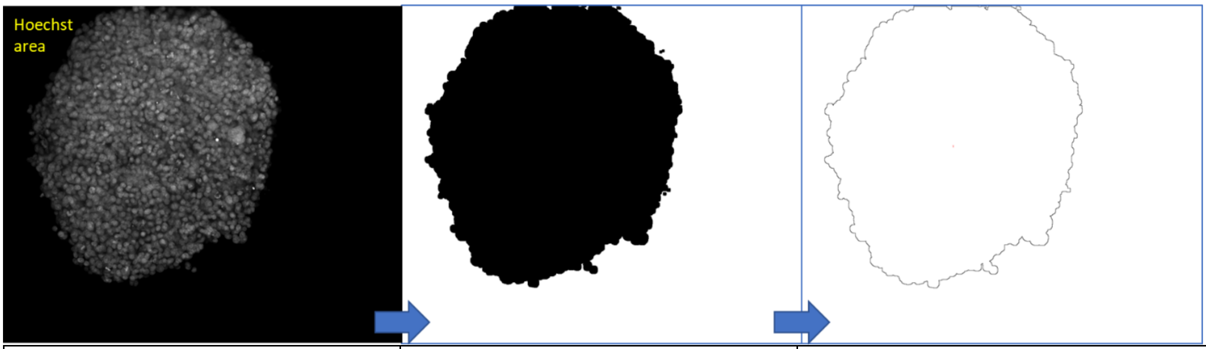

Max Int Projection from all images per channel per spheroid per well<br>No image correction<br>setAutoThreshold("Huang dark")<br>run("Convert to Mask");	run("Fill Holes");<br>run("Dilate");	Measure particle size<br>= spheroid size

CC3 and Ki67 biomarkers. CC3 signal is cytoplasmic while the Ki67 signal is nuclear. However, fluorescent labelling for these two biomarkers, despite localization, are consistent with treatment effects. Particle detection is pointless for CC3 as the signal is diffuse in the cytoplasm. Particle count for Ki67 will be tricky as well, while the signal is not well organized as foci (like γH2Ax).

Therefore, CC3 biomarker was quantified by a simplified method, by measuring the total positive (fluorescent) area within the spheroid and by normalizing this value with the total Hoechst area (see next montage). Ki67 was quantified by the same method but without normalization by the spheroid area, while the signal is present only in proliferating cells.

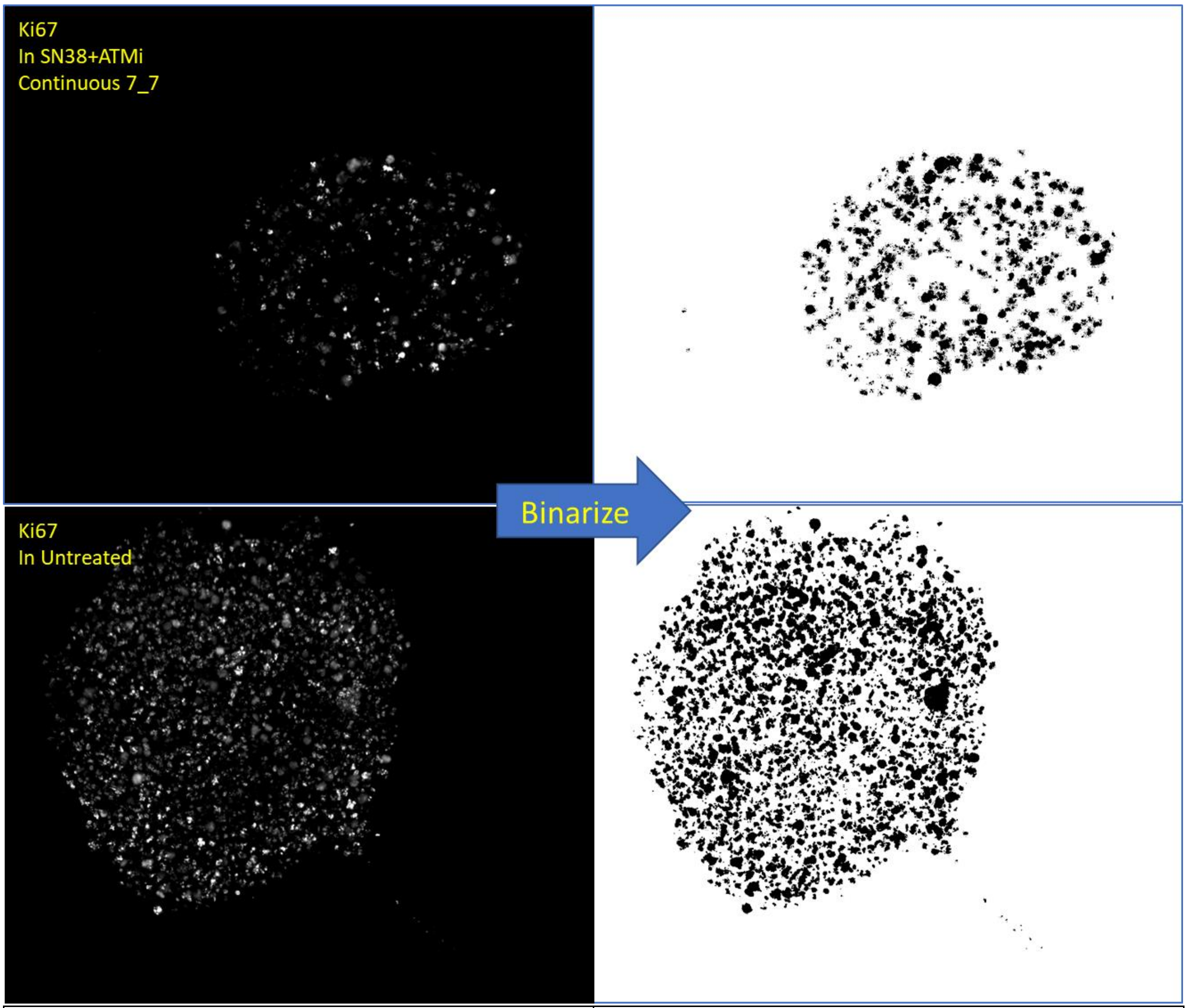

Max Int Projection from all images per channel per spheroid per well<br>No image correction<br>setAutoThreshold("Huang dark")<br>run("Convert to Mask");	Measure total stained area<br>Normalize with Total Hoechst area

All Fiji ImageJ macros used in brightfield and fluorescence image processing are attached separately.

Supplementary Method 1. Image processing for bright-field images

Brightfield (BF) images were processed in Fiji ImageJ, using a macro script derived from the macro developed by William J Ashby, Vanderbilt University. The macro is following the following steps:

1. Set scale corresponding to hardware image-embedded Leica microscope scale;
2. Convert images to 8-bit and binarize;
3. Each binarized spheroid projection image is dilated, remaining holes are filled in, then the projection image is shrunk back, using the same radius factor as for dilation step; these operations are filling the potential holes in the binarized image, due to spheroid inhomogeneities, maintaining the original spheroid size.
4. Define a min/max particle size for the spheroids;
5. Analyse particles within a circularity range of 0.2-1, to detect particle area, radius, min/max feret diameters;
6. Spheroid volume was evaluated following radius deduction from the measured area.

ImageJ macro for brightfield images

```
run("Set Scale...", "distance=53 known=100 unit=um global");
run("8-bit");
setOption("BlackBackground", false);
run("Convert to Mask", "method=IJ_IsoData background=Light");
run("Median...", "radius=2");
run("Fill Holes");
run("Watershed");
run("Analyze Particles...", "size=50000-Infinity show=Outlines display exclude summarize in_situ");
```

Supplementary Figure 3. Spheroid processing and antibody labelling for immunofluorescence

For antibody labelling, spheroids were processed in the chip. The protocol included 4 steps:

1. **Step 1** – spheroid washing, fixing, permeabilization and non-specific binding block. This step used the OB1 MK3 controller (pump), at a flow rate of 80µL/min;
2. **Step 2** – antibody labelling. Diluted antibodies are mixed with Hoechst 33342 solution (1:5000) and loaded in the chip manually, using a 1000µL pipette/tips. Next, Luer ports on the Ibidi chip are covered by polypropylene caps (provided with the Ibidi chip); the chip was incubated overnight, in the cold room at 4°C on a rocking plate.
3. **Step 3** – washing step to remove antibody excess
4. **Step 4** – prepare imaging step – remove polymer coverslip from Ibidi chip, spheroid removal and transfer in the Corning 7007 ultra-low adherence plate 96-well plate, one spheroid per well;

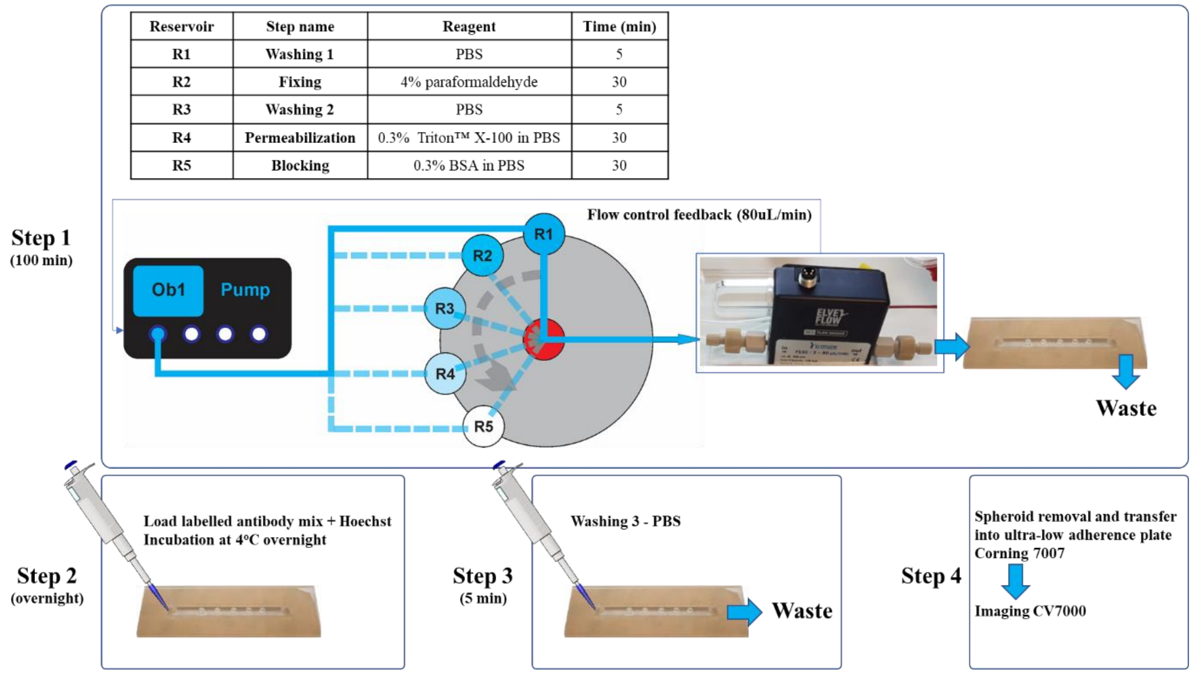

Reservoir	Step name	Reagent	Time (min)
R1	Washing 1	PBS	5
R2	Fixing	4% paraformaldehyde	30
R3	Washing 2	PBS	5
R4	Permeabilization	0.3% Triton™ X-100 in PBS	30
R5	Blocking	0.3% BSA in PBS	30

Supplementary Figure 4. Cleaved caspase-3 (CC3) biomarker detection and quantitation in SW620 spheroids at 7 days of treatment, following 5 different treatment schedules

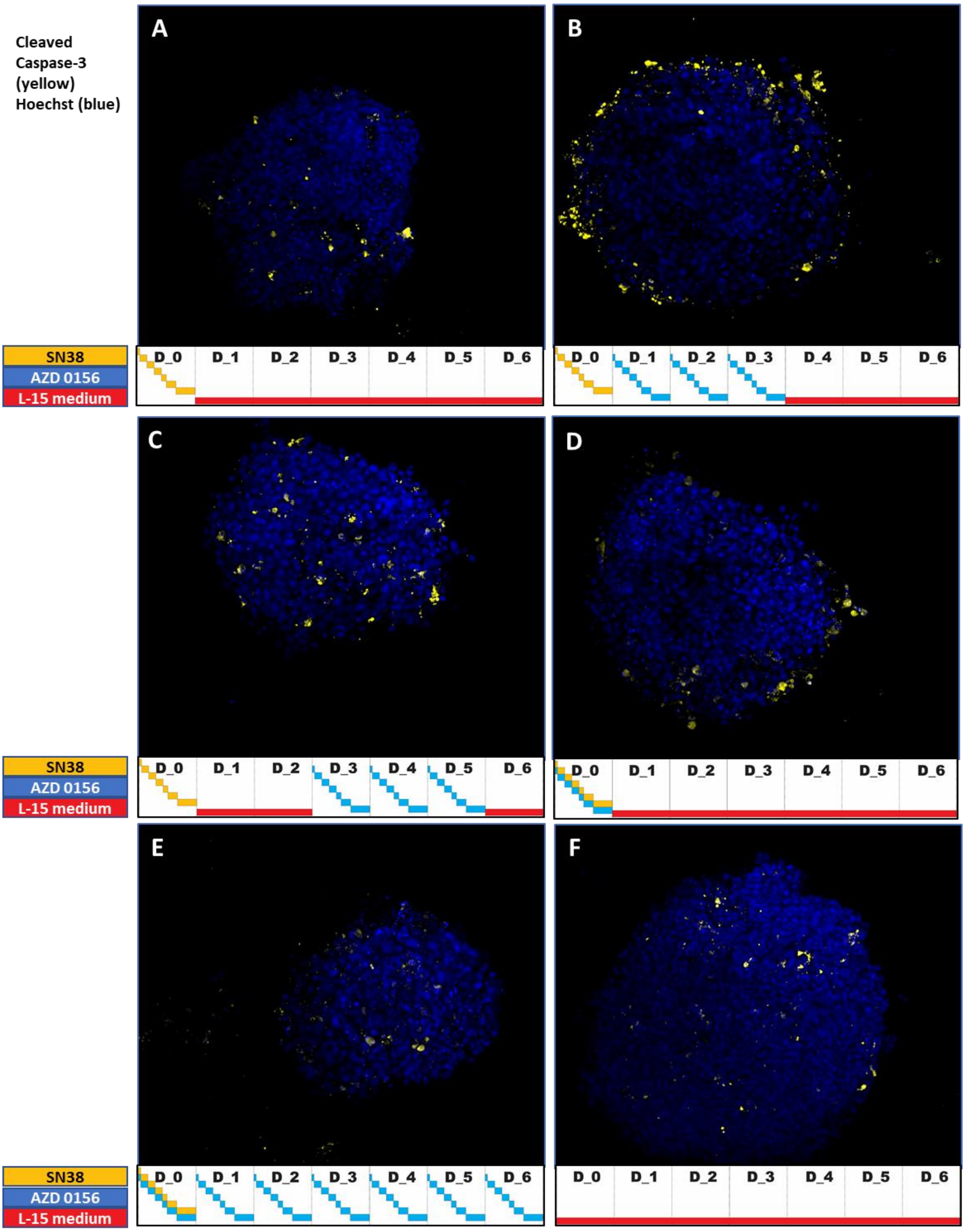

Endpoint (7 days) CC3 (yellow) in spheroids following 5 different treatment schedules. (A) SN38 monotherapy; (B) SN38+AZD0156 3/7 (with a 24h gap); (C) SN38+AZD0156 3/7 (with a 72h gap); (D) SN38+AZD0156 1/7; (E) SN38+AZD0156 7/7; (F) Control.
*All treatment schedules were tested in the microfluidic setup to generate an 8-step PK profile for both tested compounds, as described in Figure 2a,b and Supplementary S5.

Supplementary Figure 5. Ki67 biomarker detection and quantitation in SW620 spheroids at 7 days of treatment, following 5 different treatment schedules

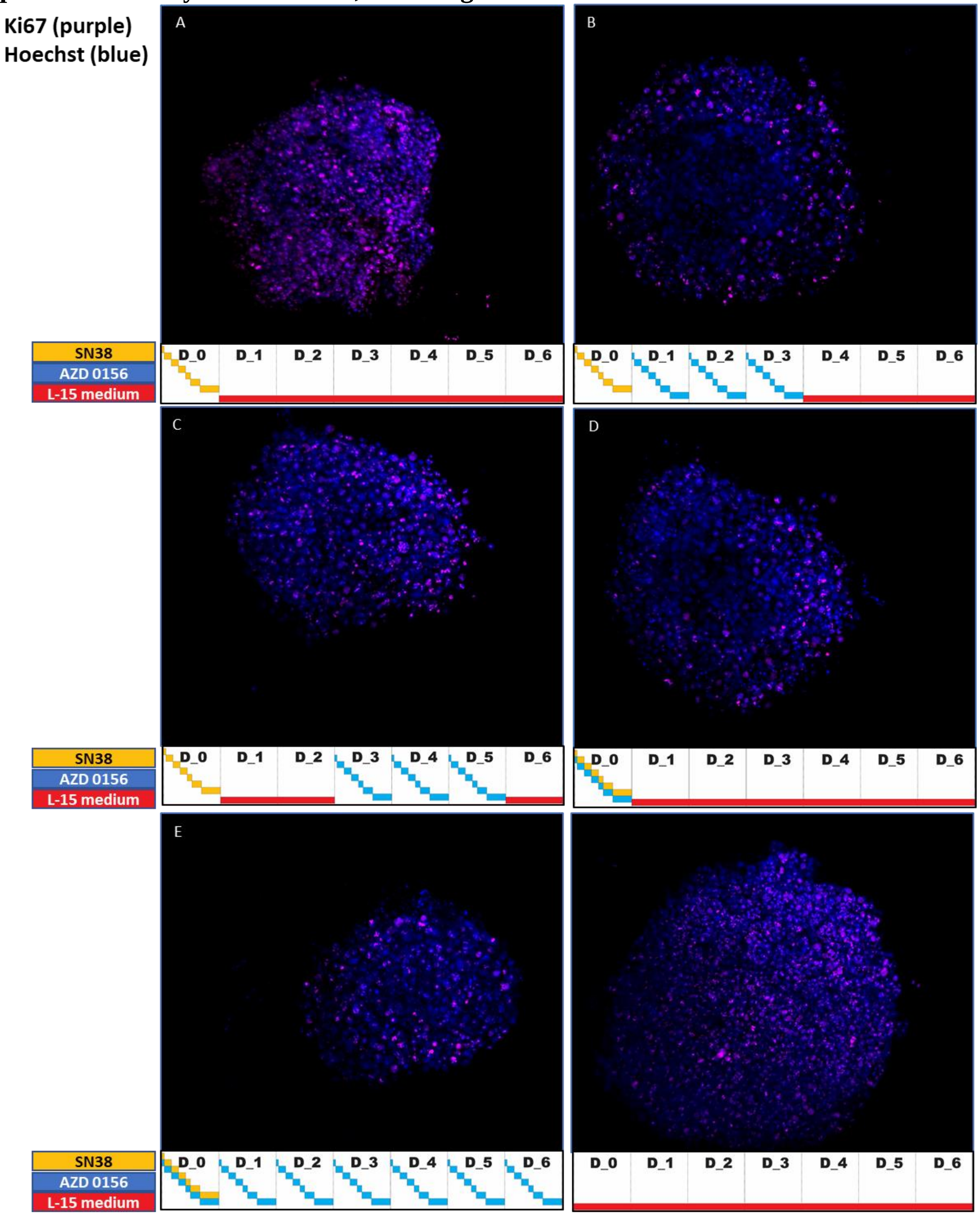

Endpoint (7 days) Ki67 (magenta) in spheroids following 5 different treatment schedules. (A) SN38 monotherapy; (B) SN38+AZD0156 3/7 (with a 24h gap); (C) SN38+AZD0156 3/7 (with a 72h gap); (D) SN38+AZD0156 1/7; (E) SN38+AZD0156 7/7; (F) Control.
*All treatment schedules were tested in the microfluidic setup to generate an 8-step PK profile for both tested compounds, as described in Figure 2a,b and Supplementary S5.

Supplementary Figure 6. % Tumour/Spheroid Growth Inhibition comparison in vivo/in vitro MF setup

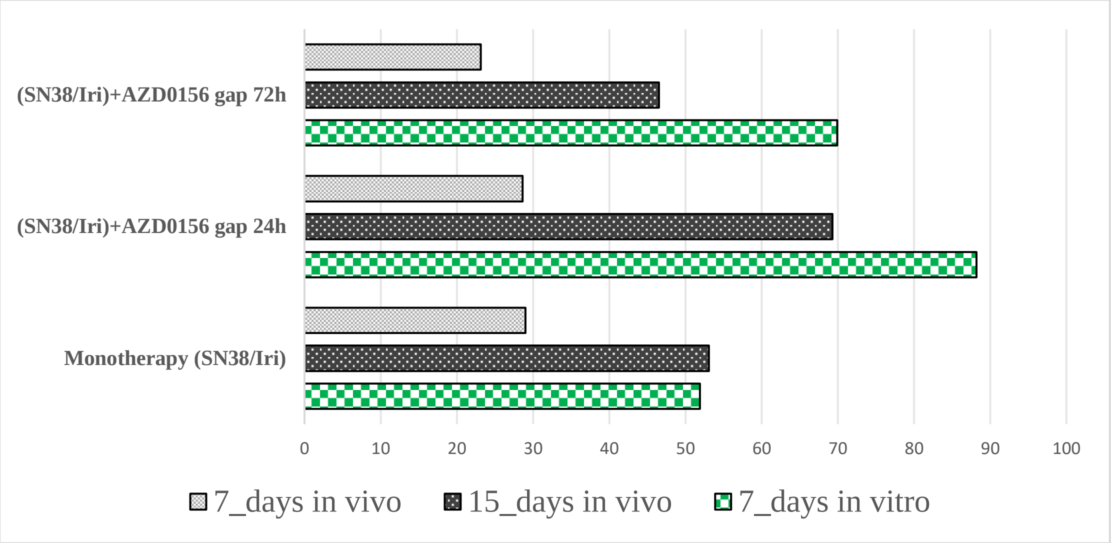

Supplementary Method 2. Spheroid transfer and encapsulation in Ibidi chip.
For a safe spheroid transfer and encapsulation in the single channel Ibidi chip, several steps were observed:

1. At 48h from cell dispensing, fully formed spheroids were individually picked-up from the wells of the 96-well low adherence plates using a 100uL pipette set to 5uL and 200µL tips; as spheroid collection can be tedious, a pair of magnifying glasses (Head Mounted Head Magnifying Glasses, Yoctosun) with 2.5x lenses has been used. For better spheroid spotting, a matte white sheet placed behind the focal plane (plate's bottom) is helpful.
2. Each 5µL of the well media including the picked spheroid were discarded on the lid of a 100mm Petri dish (P5606, Sigma Aldrich), keeping the pipette tip 1mm away from the lid. 10 droplets each containing single spheroid were formed for each condition (Ibidi chip).
3. Phenol-free Matrigel (354248, Corning) is used for encapsulation. Matrigel should be thawed on ice within 1h prior encapsulation protocol onset. 5µL Matrigel are loaded in a 200µL pipette tip using the same 100µL pipette. The 200µL tips were preferred to the 20µL ones due to wider bore that avoids spheroid squeezing. Wide bore opening 200µL tips were also considered but the volume to be manipulated is too low for a thorough reproducible Matrigel droplet formation in the Ibidi chip. The tip loaded by 5µL cooled Matrigel is immediately mixed with the one spheroid-containing droplet on the Petri dish lid. Then, 5µL mixture from the droplet, including the spheroid, are aspirated in the same tip and spheroid is slowly discarded on the ceiling (top inside part) of the open-bottom Ibidi chip. To perform this operation, Ibidi chip must be flipped upside-down, to expose the top inner side of the channel. We recommend to perform this spheroid transfer into the chip using 2.5x magnifying glasses. As spheroid comes out from the tip, Matrigel dispensing can be stopped. It is important to get the droplets with similar sizes. As the droplet volume is supposed to be around 1-1.5µL in order for the spheroid height not to exceed the Ibidi chip channel depth, this droplet dispensing procedure may be tedious but with a decent learning curve.
4. After dispensing 8 evenly-spaced spheroids on the top inner side of the Ibidi chip channel, chips were flipped back to the normal position, placed in a sterile lidded Petri dish and incubated for 5-10 min in a 37°C incubator. It's important to place the Petri dish on the bottom shelf to minimize evaporation. This step is required for Matrigel gelation and spheroid encapsulation. The Matrigel droplet is adherent to chip's ceiling and will not be removed by culture media flow. It is important to perform Matrigel gelling prior to bottom coverslip attachment, while liquid droplets might be lost, contaminated or smeared during coverslip manipulation.
5. Following Matrigel gelation, Ibidi chips are removed from the incubator and transferred in the class-2 cabinet; the liner is removed from the chip's sticky bottom and the corresponding cut-to size polymer coverslip is aligned and attached. Uniform pressure is applied to the film for full adhesion to the chip's bottom using the Ibidi clamp for sticky slides (80040, Ibidi) and the corresponding adapter for sticky slide Luer (80042, Ibidi).
6. Following sealing procedure, chip is filled with about 200µL media and the Luer ports capped with the provided sterile polymer caps. Do not overfill the chip so the Luer ports will not spill at the interface with the corresponding caps. Chips are now ready to be connected to corresponding Luer connectors of each microfluidic path.
7. For convenience and ease of manipulation, Ibidi chips are contained in a custom-made slide frame (to handle more than 4 chips at a time).

Supplementary Method 3. Spheroid treatment setup and schedules

To accurately place the microfluidic PK profile setup efficacy between static 3D cultures and in vivo setup, different type of constructs has been compared.

Microfluidic setup used mimicked 8-step PK profile for both tested compounds, using a 1-day SN38 monotherapy and 1-day/7-day schedule for SN38/ATMi combo-therapy, respectively.

Next, efficacy of monotherapy versus combo-therapy in MF setup only was evaluated for a 7-day cycle. For combo-therapy, different schedules have been assessed, consistent with in vivo data.

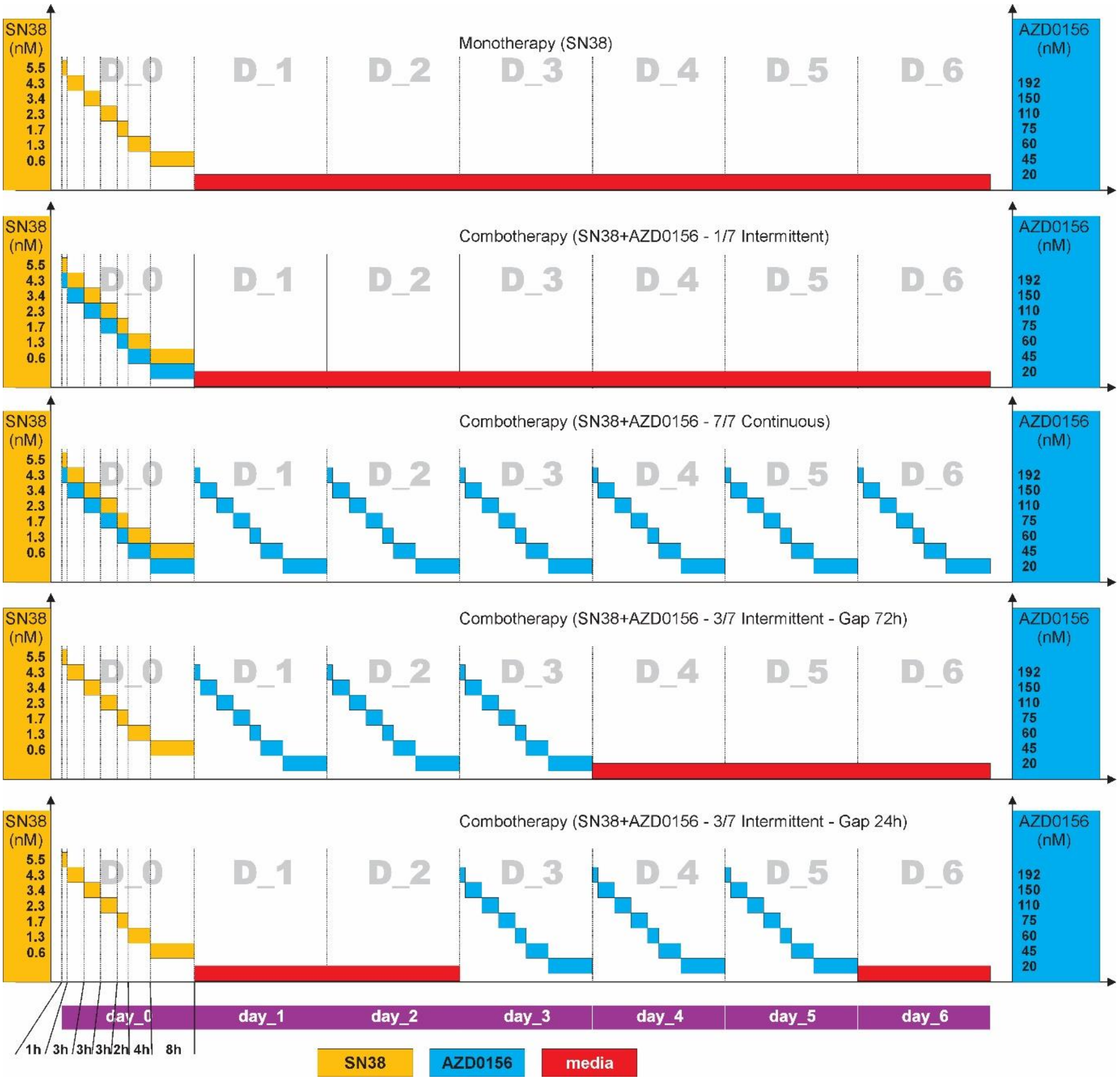

Supplementary Method 4. Translate in vivo plasma PK profiles for SN38 and AZD0156 to in vitro experimental setup, for microfluidic platform

Free SN38 and AZD0156 concentrations in mouse were measured hourly for 96h, using Mass Spec methods. In vitro experimental setup used 8 concentration points translated from the 24 hours plasma concentrations in mouse. Each concentration used for a specific amount of time for in vitro microfluidic setup represented an average of the corresponding time interval from in vivo data.

For example, in vivo average measured concentration for SN38 was of 4.36nM, recorded between 2-4h from treatment onset; in vitro microfluidic setup used a concentration of 4.3nM, for 3h, for the corresponding time interval.

Closely related PK profiles between mouse plasma and in vitro concentrations are illustrated in Figure 2b.

Supplementary Method 5. Mass Spectrometry Bioanalysis

Mass Spectrometer and UPLC system parameters

**Mass Spec**	Waters Xevo TQS		
**UPLC system**	Water s Acquity i-Class		
**Column**	Phenomenex Kinetix C18 50 x 2.1, 1.7u		
**Solvent A**	95% Water, 5% MeOH + 0.1% Formic acid		
**Solvent B**	95% MeOH, 5% Water + 0.1 % Formic acid		
**Gradient**	Time (min)	**% A**	**%B**
	0	95	5
	0.3	95	5
	2.2	5	95
	2.6	5	95
	2.61	95	5
	2.8	95	5
**Flow**	0.6 ml/min		
**Run time**	2.8 min, use a divert valve for initial 0.5 minutes		

Optimization parameters for mass spectrometry analysis

**Compound**	**Ionization mode**	**Polarity**	**Parent ion**	**Daughter ion**	**Cone voltage (v)**	**Collison Energy**	**Retention Time Min**
AZD0156	ESI	Positive	462.172	86.075	50	22	1.25
SN38	ESI	Positive	393.206	249.135	70	52	1.63
AZ10024306 (ISTD)	ESI	Positive	405.11	174.23	70	22	1.61

Compound detection in reservoirs and at the flow path outlet

AZD0156 (nM)				
Planned conc	Reservoir conc		Outlet conc	At time (h)
192	r2	258.3	225.2	1
150	r3	127.3	125.4	4
110	r4	93.8		7
75	r5	72.6		10
60	r6	59		12
45	r7	40.3		16
20	r8	21.5	17	24

SN38 (nM)					
Planned conc	Reservoir conc		Outlet conc	At time (h)
5.5	r2	9.8	7.1	1
4.3	r3	5	5.9	4
3.4	r4	4.2		7
2.3	r5	3.8		10
1.7	r6	2.2		12
1.3	r7	1.9		16
0.6	r8	0.8	<0.0025	24

Supplementary Method 6. Compound dilution

In microfluidic (MF) setup, volumes were calculated considering the flow rate/time for the fluid in each reservoir. A 2-step dilution was considered for the MF setup, due to relatively large volumes required in the reservoirs (10-40mL).
A first step dilution for SN38 (from 0.1mM stock to 1µM sub-stock) and AZD0156 (from 1mM stock to 19.2µM sub-stock) was performed, using a HP D300e Digital Dispenser (Hewlett-Packard) and a flat-bottom 96-well plate. Thorough mixing in the plate wells must be considered.
The second dilution step was performed manually, considering the final volumes to be used in the main flow reservoirs (see tables below), for a 7-day cycle, mono or combo-therapy. Thorough mixing in the 50mL falcons (reservoirs) must be considered.

**SN38**	**stock 0.1mM / 1st dilution 1µM**						
Reservoir label	Res.2	Res.3	Res.4	Res.5	Res.6	Res.7	Res.8
Flow time (h)	1	3	3	3	2	4	8
Working concentration (µM)	5.5	4.3	3.4	2.3	1.7	1.3	0.6
Required volume (mL) for 1 day	1.2	3.6	3.6	3.6	2.4	4.8	9.6
**Final volume per reservoir (mL)**	**10**	**10**	**10**	**10**	**10**	**10**	**20**
**SN38 (1µM) volume (µL) per reservoir**	**55**	**43**	**34**	**23**	**17**	**13**	**12**

**AZD0156**	**stock 1mM / 1st dilution 19.2µM**						
Reservoir label	Res.2	Res.3	Res.4	Res.5	Res.6	Res.7	Res.8
Flow time (h)	1	3	3	3	2	4	8
Working concentration (µM)	192	150	110	75	60	45	20
	**1-day intermittent schedule**						
Required volume (mL) for 1 day	1.2	3.6	3.6	3.6	2.4	4.8	9.6
**Final volume per reservoir (mL)**	**10**	**10**	**10**	**10**	**10**	**10**	**20**
**AZD0156 (19.2µM) volume (µL) per reservoir**	**100**	**78**	**57**	**39**	**31**	**23**	**21**
	**3-day intermittent schedule (24 and 72h gap)**						
Required volume (mL) for 3 days	3.6	10.8	10.8	10.8	7.2	14.4	28.8
**Final volume per reservoir (mL)**	**10**	**20**	**20**	**20**	**20**	**30**	**40**
**AZD0156 (19.2µM) volume (µL) per reservoir**	**100**	**157**	**115**	**79**	**63**	**71**	**42**
	**7-day continuous schedule**						
Required volume (mL) for 7 days	8.4	25.2	25.2	25.2	16.8	33.6	67.2
**Final volume per reservoir (mL)**	**20**	**30**	**30**	**30**	**30**	**30**	**80***
**AZD0156 (19.2µM) volume (µL) per reservoir**	**200**	**234**	**172**	**117**	**94**	**70**	**31**

* - use a 100mL glass bottle with 4 port cap (Darwin Microfluidics)

Supplementary Method 7. Image processing for immunofluorescence images

As described in main text, spheroids in the microfluidic Ibidi chips were labelled for γ-H2Ax, CC3 and Ki67 and then transferred in a Corning 7007 ultra-low adherence plate 96-well plate, containing one spheroid per well. To detect these biomarkers, images were generated on a CV7000 confocal microscope. Wells on the Corning 7007 plate, containing one spheroid per well, were imaged individually, generating a corresponding number of folders with image batches. Each image batch was comprised in a folder containing:

- a file with extension *.ppp, describing machine specific measurement parameters;
- a file with extension *.wpi that allow Fiji ImageJ to open files in the batch/folder according to each fluorescence channel;
- all raw images in the batch, for all spheroids in the plate, all channels and all slices/spheroid, in tiff format.

Opening image batch in Fiji ImageJ

From each folder corresponding to the image batch (1 batch/spheroid), the *.wpi file has to be dragged and dropped to the opened Fiji ImageJ interface.

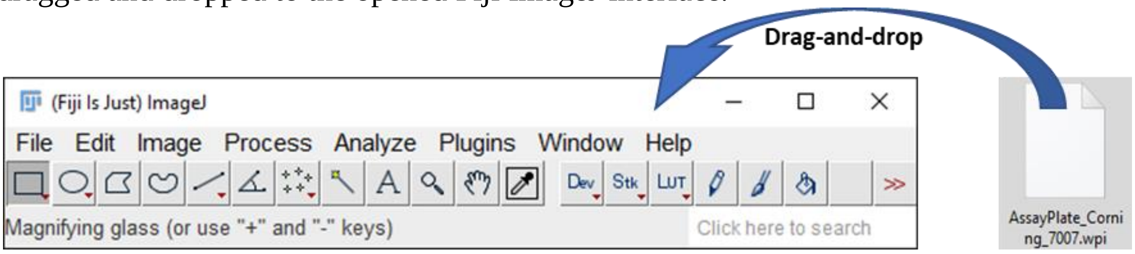

The bioformat options window opens, and the following options will be checked:

- Stack viewing – as hyperstack;
- Dataset organization – please check:
    - Group files with similar names;
    - Open files individually;
- Memory management – please check:
    - Use virtual stack;
- Split into separate windows – please check:
    - Split channels;

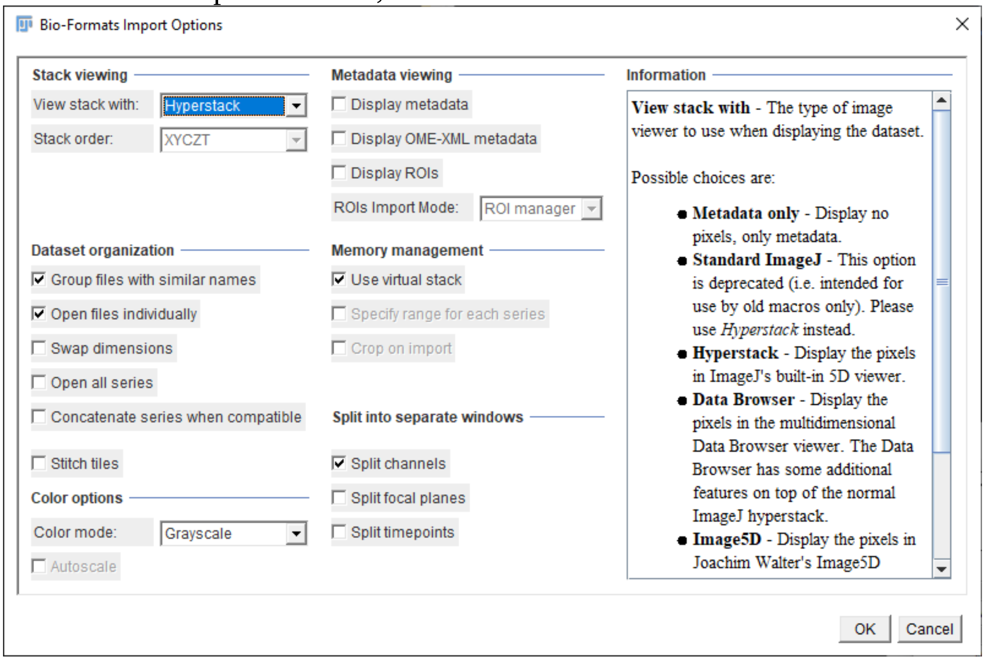

As a result of checking these options, a set of 4 windows are being displayed, each window representing the image batch for one spheroid, for each of the 4 fluorescence channels.
Our protocol explored 4 fluorescence channels, using the 20x objective:

Wavelength	Biomarker	Fluorophore	Pseudo-colour	Channel no.
676-29	Ki67	Alexa Fluor 647	Cyan	0
445-45	Nucleus	Hoechst	Blue	1
600-37	CC3	Alexa Fluor 594	Yellow	2
525-50	γH2Ax	Alexa Fluor 488	Green	3

Macro code for blue channel

```
run("8-bit");
setAutoThreshold("Huang dark");
run("Convert to Mask");
run("Maximum...", "radius=2");
run("Fill Holes");
run("Dilate");
run("Fill Holes");
run("Dilate");
run("Analyze Particles...", "size=70000-Infinity show=Outlines display summarize");
```

Macro code for green channel

```
run("Z Project...", "projection=[Max Intensity]");
run("8-bit");
run("Subtract Background...", "rolling=20");
run("Set Measurements...", "area mean standard integrated area_fraction stack display add
redirect=None decimal=1");
setOption("BlackBackground", false);
run("Convert to Mask");
run("3D Objects Counter", "threshold=128 slice=1 min.=15 max.=50 objects statistics
summary");
```

Supplementary Method 8. Pharmacokinetic models parameters used for simulations

AZD156 PK model and PK parameters used for simulations

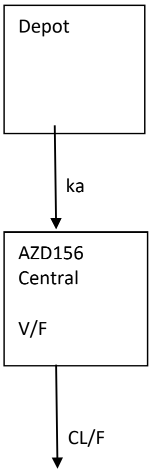

Parameter (units)	Mean	Std error	%RSE
ka (hr^{-1})	1.4	0.322	23
Vd/F Volume of distribution (L)	23.3	0.254	1.2
Clearance (L/kg)	5.03	0.142	2.8

Colour lines represents observed data from 3 mice, black line model fitted based on PK model.

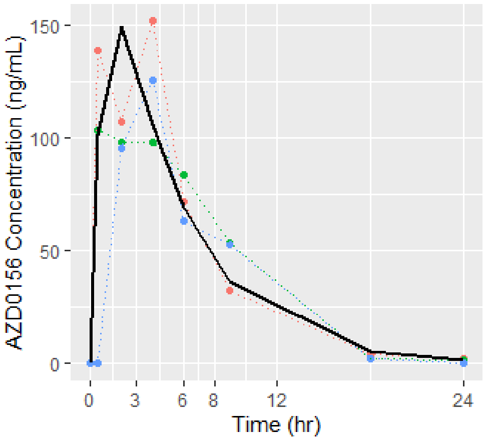

Irinotecan PK model and PK parameters used for simulations

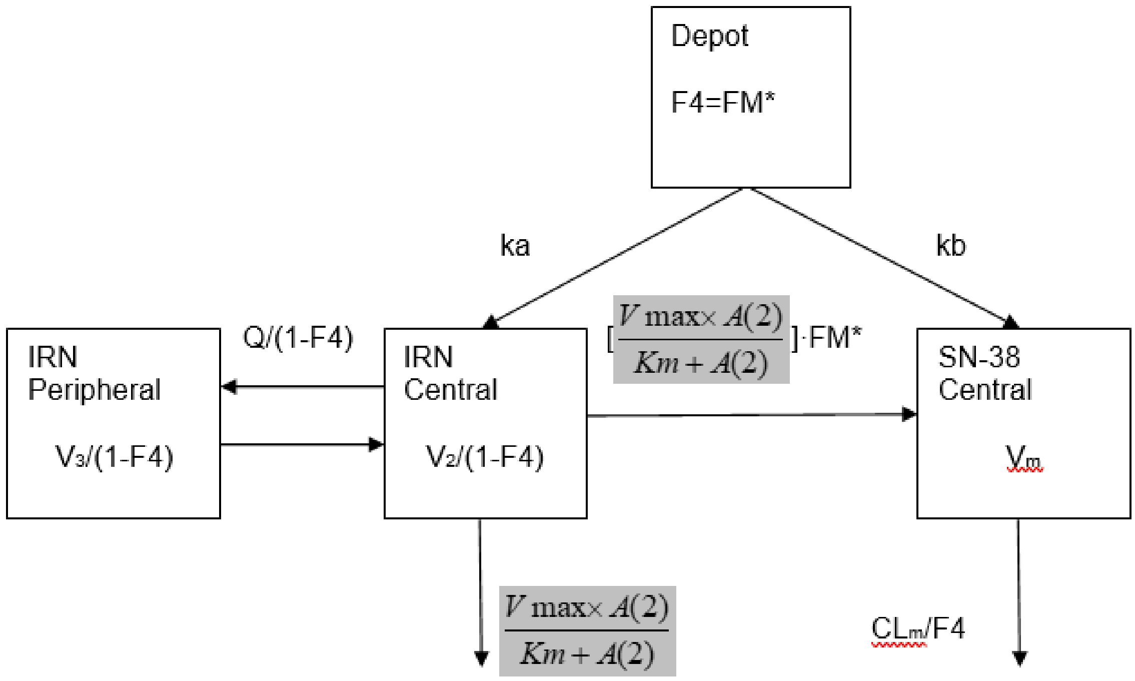

Parameter (units)	Mean	Std error	%RSE
ka (hr^{-1})	0.454	0.085	18.7
kb (hr^{-1})	100 (fixed)		
FM*	0.286	0.0553	19.3
Vmax (μM/hr)	0.0589	0.0686	116
Km (μM)	0.00612	0.00612	100
V_2/(1-F4) (L)	0.0161	0.00644	40
Q/(1-F4) (L/hr)	0.0574	0.0187	32.6
V_3/(1-F4) (L)	5.26	5.10	97.0
CL_m/F4 (L/hr)	0.220	0.0479	21.8
ω^2 [V_2/(1-F4)]	0.126	0.245	194
ω^2 (FM*)	0.348	0.241	69.3
Residual error, IRN (μM)	0.433	0.0814	18.8
Residual error, SN38 (μM)	0.344	0.0626	18.2

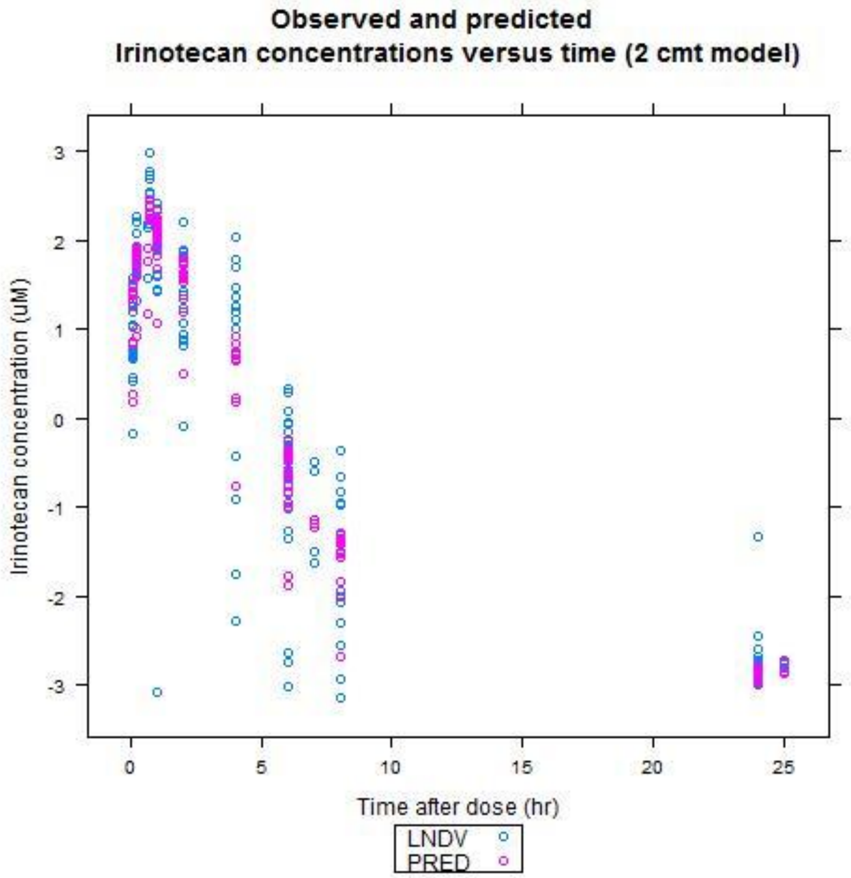

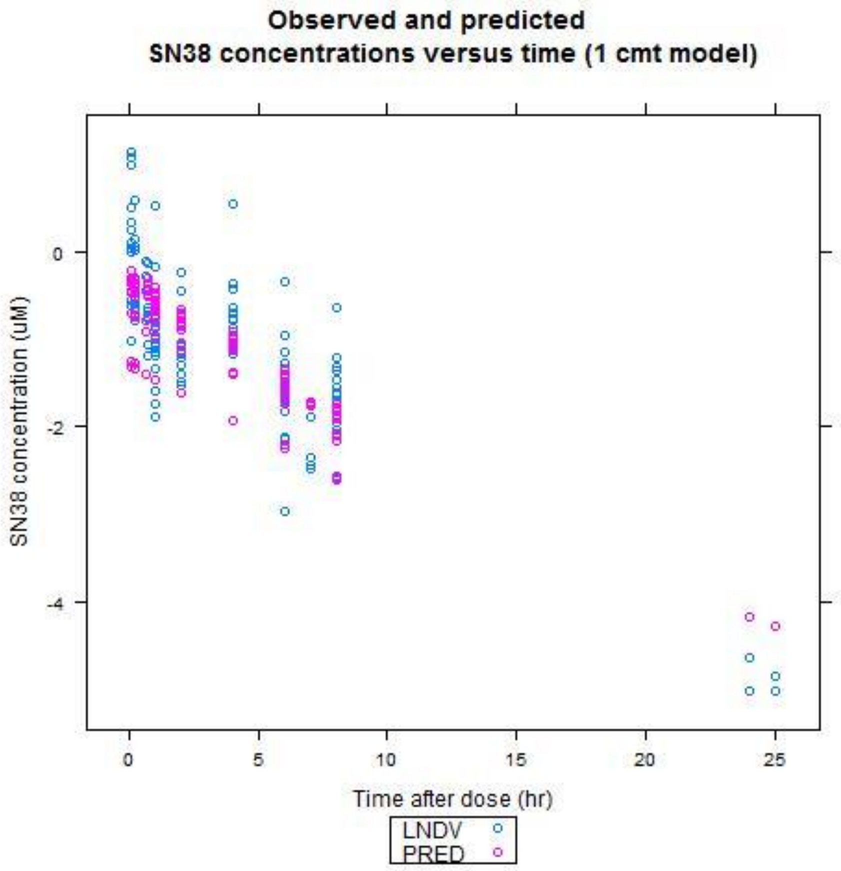

Blue circles represent the observed data and pink circles model fitted based on PK model.
