# Supplementary material for: Tumour-on-chip microfluidic platform for assessment of drug pharmacokinetics and treatment response
Source: Commun Biol. 2021 Aug 24;4:1001. doi: 10.1038/s42003-021-02526-y (PMC8385015; doi:10.1038/s42003-021-02526-y)
Supplement: Supplementary file 3 — Description of Additional Supplementary Files [file 42003_2021_2526_MOESM3_ESM.pdf]

Description of Additional Supplementary Files

File Name: Supplementary Data 1
Description: Source data for graphs
