# Supplementary material for: Tumour-on-chip microfluidic platform for assessment of drug pharmacokinetics and treatment response
Source: Commun Biol. 2021 Aug 24;4:1001. doi: 10.1038/s42003-021-02526-y (PMC8385015; doi:10.1038/s42003-021-02526-y)
Supplement: Supplementary file 5 — Reporting Summary [file 42003_2021_2526_MOESM5_ESM.pdf]

nature research

Corresponding author(s): DBPR COMMSBIO-20-3593

Last updated by author(s): Jun 2, 2021

Reporting Summary

Nature Research wishes to improve the reproducibility of the work that we publish. This form provides structure for consistency and transparency in reporting. For further information on Nature Research policies, see our Editorial Policies and the Editorial Policy Checklist.

Statistics

For all statistical analyses, confirm that the following items are present in the figure legend, table legend, main text, or Methods section.

n/a	Confirmed	
☐	☒	The exact sample size (*n*) for each experimental group/condition, given as a discrete number and unit of measurement
☐	☒	A statement on whether measurements were taken from distinct samples or whether the same sample was measured repeatedly
☐	☒	The statistical test(s) used AND whether they are one- or two-sided<br>*Only common tests should be described solely by name; describe more complex techniques in the Methods section.*
☐	☒	A description of all covariates tested
☒	☐	A description of any assumptions or corrections, such as tests of normality and adjustment for multiple comparisons
☐	☒	A full description of the statistical parameters including central tendency (e.g. means) or other basic estimates (e.g. regression coefficient) AND variation (e.g. standard deviation) or associated estimates of uncertainty (e.g. confidence intervals)
☒	☐	For null hypothesis testing, the test statistic (e.g. *F*, *t*, *r*) with confidence intervals, effect sizes, degrees of freedom and *P* value noted<br>*Give P values as exact values whenever suitable.*
☒	☐	For Bayesian analysis, information on the choice of priors and Markov chain Monte Carlo settings
☒	☐	For hierarchical and complex designs, identification of the appropriate level for tests and full reporting of outcomes
☒	☐	Estimates of effect sizes (e.g. Cohen's *d*, Pearson's *r*), indicating how they were calculated

Our web collection on statistics for biologists contains articles on many of the points above.

Software and code

Policy information about availability of computer code

Data collection	Prism 8, GraphPad Software, Inc., La Jolla, CA
Data analysis	Prism 8, GraphPad Software, Inc., La Jolla, CA<br>Fiji ImageJ opensource win x64

For manuscripts utilizing custom algorithms or software that are central to the research but not yet described in published literature, software must be made available to editors and reviewers. We strongly encourage code deposition in a community repository (e.g. GitHub). See the Nature Research guidelines for submitting code & software for further information.

Data

Policy information about availability of data

All manuscripts must include a data availability statement. This statement should provide the following information, where applicable:
- Accession codes, unique identifiers, or web links for publicly available datasets
- A list of figures that have associated raw data
- A description of any restrictions on data availability

The authors declare that [the/all other] data supporting the findings of this study are available within the paper [and its supplementary information files].

Field-specific reporting

Life sciences study design

All studies must disclose on these points even when the disclosure is negative.

Sample size	For in vivo study, n=15 mice per treatment group were used in the efficacy study and n=3 mice per timepoint were used to determine the compounds PK profile. For the in vitro experiments, n=5-6 spheroids per treatment group were used.
Data exclusions	Not applicable
Replication	For in vitro studies, each treatment condition was repeated at least 2-3 times and measurements for each condition were pooled from the replicates.
Randomization	Not applicable
Blinding	Not applicable

Reporting for specific materials, systems and methods

We require information from authors about some types of materials, experimental systems and methods used in many studies. Here, indicate whether each material, system or method listed is relevant to your study. If you are not sure if a list item applies to your research, read the appropriate section before selecting a response.

Materials & experimental systems

n/a	Involved in the study	
☐	☒	Antibodies
☐	☒	Eukaryotic cell lines
☒	☐	Palaeontology and archaeology
☐	☒	Animals and other organisms
☒	☐	Human research participants
☒	☐	Clinical data
☒	☐	Dual use research of concern

Methods

n/a	Involved in the study	
☒	☐	ChIP-seq
☒	☐	Flow cytometry
☒	☐	MRI-based neuroimaging

Antibodies

Antibodies used	primary conjugated antibodies for γH2Ax (gamma H2AX [p Ser139] Antibody [Alexa Fluor 488], NB100-384AF488, Novus Bio/BioTechne), caspase-3 (CC3, Caspase-3 Antibody (31A1067) [Alexa Fluor 594], NB100-56708AF594, and KI67 (Anti-Ki67 antibody [EPR3610] (Alexa Fluor 647), ab196907, Abcam)
Validation	γH2Ax (gamma H2AX [p Ser139] Antibody [Alexa Fluor 488]; Name: NB100-384AF488, Polyclonal rabbit IgG; Reactivity Hu, Mu, Rt, Ca; Applications WB, Flow, ICC/IF, IHC, IHC-Fr, IHC-P, KO; Conjugate Alexa Fluor 488 Novus Bio/BioTechne , https://www.novusbio.com/products/gamma-h2ax-antibody_nb100-384af488#datasheet<br>caspase-3 (CC3, Caspase-3 Antibody (31A1067) [Alexa Fluor 594], Name: NB100-56708AF594, Monoclonal mouse IgG1 kappa, Reactivity Hu, Mu, Rt, Po, ChHa, Ma, Applications WB, Flow, IB, ICC/IF, IHC, IHC-Fr, IHC-P, IHC-FrFl, KO, Clone 31A1067 https://www.novusbio.com/products/caspase-3-antibody-31a1067_nb100-56708af594<br>KI67 (Anti-Ki67 antibody [EPR3610] (Alexa Fluor 647), ab196907, Abcam), Rabbit monoclonal [EPR3610], Suitable for: ICC, Knockout validated, Reacts with: Human https://www.abcam.com/alexa-fluor-647-ki67-antibody-epr3610-ab196907.html

Eukaryotic cell lines

Policy information about cell lines

Cell line source(s)	SW620 (CCL-227, ATCC)
Authentication	No further authentication apart from ATCC original
Mycoplasma contamination	Mycoplasma negative
Commonly misidentified lines (See ICLAC register)	Bic-1, Boonstra et al. refer to the contaminant as SW620. However, SW-480 and SW-620 were derived from the same individual, so both carry the same identity; the contaminating cell line could be either of these two cell lines.

Animals and other organisms

Policy information about studies involving animals; ARRIVE guidelines recommended for reporting animal research

Laboratory animals	Swiss athymic nu/nu male mice adults

	CB-17 SCID female mice , adults
Wild animals	The study did not involved wild animals
Field-collected samples	The study did not involved samples collected from the field
Ethics oversight	All in vivo studies complied with all relevant ethical regulations for animal testing and research, followed AstraZeneca's global bioethics policy and received ethical approval from the AstraZeneca ethical committee. All studies were conducted in the UK in accordance with UK Home Office legislation, the Animal Scientific Procedures Act 1986 and under Home Office project licence 40/8894.

Note that full information on the approval of the study protocol must also be provided in the manuscript.
